# Global monkeypox case hospitalisation rates: A rapid systematic review and meta-analysis

**DOI:** 10.1016/j.eclinm.2022.101710

**Published:** 2022-10-31

**Authors:** Michael E. DeWitt, Christopher Polk, John Williamson, Avinash K. Shetty, Catherine L. Passaretti, Candice J. McNeil, Robert T. Fairman, Mindy M. Sampson, Cynthia Dalton, John W. Sanders

**Affiliations:** a Section on Infectious Diseases, Department of Internal Medicine, Wake Forest University School of Medicine, Winston–Salem, NC, USA; b Center for the Study of Microbial Ecology and Emerging Diseases, Wake Forest University School of Medicine, Winston–Salem, NC, USA; c Division of Infectious Diseases, Atrium Health, Charlotte, NC, USA; d Infectious Diseases, Department of Pediatrics, Wake Forest University School of Medicine, Winston–Salem, NC, USA; e Department of Health Policy & Behavioral Sciences, School of Public Health, Georgia State University, Atlanta, GA, USA

**Keywords:** Monkeypox, Vaccination, Surveillance, Outbreak, Hospitalization

## Abstract

**Background:**

Estimates of the case hospitalization rate and case fatality rate when hospital care is available for monkeypox (MPX) infections have not been well defined. This rapid systematic review and meta-analysis aimed to estimate the case hospitalisation rate and case fatality rate where hospital care is available.

**Methods:**

We systematically searched PubMed, Embase, the Lancet Preprints, and MedRxiv for studies published between Jan 1, 1950 and Aug 2, 2022. We included documents which contained both the number of cases and associated hospitalisations of MPX infections. From eligible studies we extracted the country, the year of the study, the study design type, the clade of MPX, the participant characteristics, transmission type, any treatments used, number of cases (including suspected, probable, or laboratory confirmed diagnosis), number of hospitalizations, hospitalized patient outcomes, and case definition. Case hospitalization rate (CHR) was defined as the proportion of cases that were admitted to hospital care while case fatality rate (CFR) was defined as the proportion of cases that died. CHR and CFR were analysed in a fully Bayesian meta-analytic framework using random effects models, including sub-group analysis with heterogeneity assessed using I^2^.

**Findings:**

Of the 259 unique documents identified, 19 studies were eligible for inclusion. Included studies represented 7553 reported cases among which there were 555 hospitalizations. Of the 7540 cases for which outcomes were available, there were 15 recorded deaths. The median age of cases was 35 years (interquartile range 28–38, n = 2010) and primarily male (7339/7489, 98%) in studies where age or sex were available. Combined CHR was estimated to be 14.1% (95% credible interval, 7.5–25.0, I^2^ 97.4%), with a high degree of heterogeneity. Further analysis by outbreak period indicates CHRs of 49.8% (28.2–74.0, I^2^ 81.4%), 21.7% (7.2–52.1, I^2^ 57.7%), and 5.8% (3.2–9.4, I^2^ 92.4%) during the pre-2017, 2017–2021, and 2022 outbreaks, respectively, again with high levels of heterogeneity. CFR was estimated to be 0.03% (0.0–0.44, I^2^ 99.9%), with evidence of large heterogeneity between the studies.

**Interpretation:**

There is limited data for MPX hospitalization rates in countries where MPX has been traditionally non-endemic until the current outbreak. Due to substantial heterogeneity, caution is needed when interpreting these findings. Health care organizations should be cognizant of the potential increase in healthcare utilization. Rapid identification of infection and use of appropriate therapies such as antivirals play a role reducing the CHR and associated CFR.

**Funding:**

None.

**Research in context**

**Evidence before this study**

We searched PubMed and Embase for manuscripts published in English from inception until Aug 2, 2022 with variations of “monkeypox” and “monkey pox” in combination with “case hospitalization” or “case hospitalisation”. We found no published meta-analysis discussing case hospitalisation rates from monkeypox infections and accordingly no published meta-analysis of case fatality rates from monkeypox where hospitalisations were recorded.

**Added value of this study**

In this meta-analysis and systematic review of 19 studies, we employ a Bayesian meta-analytic framework in order to estimate the likely case hospitalisation rate and case fatality rates where hospitalisation is available. This is the first meta-analysis to estimate case hospitalisation rates and case fatality rates where hospitalisation is available. The results from pooled estimates suggest that there has been an attenuation of the case hospitalisation rate from nearly 50% during pre-2017 outbreaks to 3.2–9.4% during the 2020 outbreak. However, all meta-analyses displayed high levels of heterogeneity indicating likely addition sources of variation that were not captured in the studies. Understanding the proportion of cases expected to require hospitalisation is important for health resource planning during outbreaks of monkeypox, especially in non-endemic countries.

**Implications of all the available evidence**

These findings can be used to inform health agencies and health systems in order to estimate the likely number of hospitalisations and suspected fatalities from those infected with monkeypox. Moreover, the high level of heterogeneity observed in this meta-analysis suggests that other potential contributing factors to case hospitalisation rate should be examined.

## Introduction

With the growing global outbreak of monkeypox (MPX) in traditionally non-endemic countries, including those in Europe and North America, the World Health Organization (WHO) Director General declared that monkeypox constituted a Public Health Emergency of International Concern (PHEIC) on July 23, 2022.^1^, ^2^, ^3^, ^4^ As of August 2, 2022, over 26000 cases of MPX have been reported worldwide since January 2022 in the current global outbreak.^3^

Monkeypox virus is a double-stranded DNA virus belonging to the *orthopoxvirus* genus, which includes smallpox, and has been considered endemic to West and Central Africa.^5^^,^^6^ Two primary distinct genetic clades of MPX have been classified (Clade I, the Congo Basin [Central Africa], and Clade II, the West African clade).^6^, ^7^, ^8^, ^9^ The animal reservoirs of MPX are unknown, but MPX has been shown to infect monkeys, prairie dogs, and other members of the rodent family.^10^, ^11^, ^12^, ^13^ Animal-to-human transmission is thought to occur from contact with the blood, bodily fluids, or mucosal lesions of infected animals.^5^ In non-endemic countries, cases related to international travel or importation of MPX-infected animals have been reported.^14^^,^^15^ Direct human-to-human transmission is likely to occur through prolonged, close contact with skin lesions or respiratory secretions of infected individuals.^5^ Fomite transmission is also possible (e.g. contaminated surfaces and bed linens).^9^ In utero transmission of MPX and foetal deaths have been reported.^16^ Early epidemiological studies have found that onward human transmission often terminates after four to six onward transmissions, with further modelling studies suggesting basic reproduction numbers less than one.^17^, ^18^, ^19^, ^20^ Basic reproduction numbers less than one would indicate a lower likelihood of sustained human-to-human transmission growing into epidemics. With the cessation of the smallpox vaccination programs however, there is a growing proportion of the population that is susceptible to MPX.^21^, ^22^, ^23^ Modelling studies have shown that dense sexual networks among men who have sex with men (MSM), particularly when there are multiple sexual partners could catalyse basic reproduction numbers above one despite relatively low secondary attack rates for MPX.^24^ A recent analysis indicates that the effective reproduction number of the current outbreak is likely above one world-wide.^25^ To date in the 2022 outbreak, the majority of reported MPX cases have been among individuals who identify as gay, bisexual, or MSM.^26^^,^^27^

As an emerging infectious disease, MPX potential for severe infections requiring hospitalization has not been well characterized.^27^, ^28^, ^29^ The case fatality rate is estimated at 10% with the Congo Basin clade and 3–6% in the West African clade.^7^^,^^9^ As the current outbreak of monkeypox in non-endemic countries continues to unfold, understanding the number of infected individuals who will likely require hospital care is important to inform intervention strategies and to prepare to adequately care for those infected with MPX. Similarly, in evaluating the case fatality rate of MPX infections, the public may better understand and adopt strategies to slow and stop transmissions. Understanding and anticipating the potential need for hospital care has been an important lesson learned during the COVID-19 pandemic, and access to adequate care can have a positive impact on case outcomes.^30^ With the growing number of MPX cases around the world, a key question remains as to what proportion of cases will ultimately require hospitalization. The aim of this study is to estimate the case hospitalization rate (CHR) for MPX cases as well as the case fatality rate (CFR) when hospitalization is available in order to better inform public health on the potential number of cases requiring hospitalization.

## Methods

This study was conducted according to the Preferred Reporting Items for Systematic Reviews and Meta-Analyses (PRISMA) statement.^31^ It was not prospectively registered and a study protocol was not prepared. This study was reviewed by the Wake Forest University School of Medicine Institutional Review Board who determined it was exempt from full review as the study does not meet the federal definition of research involving human subjects.

### Search strategy and selection criteria

On 2 August 2022, we systematically searched the PubMed and Embase databases for literature, from January 1, 1950 to August 2, 2022. Different search types and patterns were used through combinations of monkeypox (e.g., “monkeypox” OR “monkey pox”), and hospitalization (e.g., “hospital” OR “hospitalization” OR “hospitalisation”) with no language restrictions. These search criteria were also manually applied to two preprint servers: medRxiv and The Lancet Preprints. Full details on the search strategies are available in the Appendix. We made use of standard keyword searches and Boolean operators. The returned literature was enriched with bibliographic searches of the returned articles which did not appear in the database searches. Furthermore, national and international reporting agencies (i.e., World Health Organization, European Centre for Disease Prevention and Control, United States Centers for Disease Control and Prevention), were manually searched for reports on monkeypox cases and hospitalizations. Studies were deemed eligible for inclusion if cases and hospitalisations were both reported. Cases were defined as the number of people with a suspected, probable, or laboratory confirmed diagnosis of MPX infection. Hospitalization was defined as a patient admitted to inpatient hospital care for the management of MPX. Studies which did not indicate availability of hospital-based care were excluded. Duplicate documents returned from the database and manual searches were first removed. Literature involving single case reports or studies of only those patients who were hospitalized were excluded. Documents reporting on the same outbreak or the same data were removed in favour of the primary source. Documents that were unrelated to the research question, commentaries, editorials, focused solely on modelling, or did not have details on hospitalizations were removed.

Results were saved into Zotero (Corporation for Digital Scholarship) for further management. The full text of all documents was examined to inspect for inclusion against these criteria by two reviewers independently (MD and JW) with a third reviewer adjudicating as need (JS). For documents deemed eligible for inclusion, the country, the year of the study, the study design type, the clade of MPX where clade was identified, the participant characteristics (age, sex, sexual orientation where available), transmission type (suspected animal to human or human to human), any treatments used, number of cases, number of hospitalisations, the number of patient deaths, and case definition were recorded in a Microsoft Excel (Microsoft Cooperation, Redmond, WA, USA; 2016) spreadsheet independently by two authors (MD and JW). Any differences were mutually assessed and consensus values were entered. Bibliographic information was also included. In the case that any field was not mentioned in a given document, it was marked “Not specified.”

### Quality assessment

Risk of bias was assessed using assessment tool is adapted from the quality assessment tool developed by Hoy and colleagues^32^ and adapted by Werfalli and colleagues for studying prevalence in populations.^33^ This tool has a maximum of ten points (see Appendix for details). Eligible documents were independently screened by two reviewers (MD and JS) to evaluate the risk of bias. Documents with scores of ≥8 were considered to have a low risk of bias, while those with scores of 5 or lower were considered to have a high risk of bias, with the remaining studies considered moderate risk of bias.

### Outcomes

The primary outcome for the quantitative meta-analysis was the proportion of cases that were hospitalized (case hospitalization rates, CHR). A secondary outcome was the proportion of cases that expired, the case fatality rate (CFR). Sensitivity analysis by predominant clade was planned but not completed due to insufficient data.

### Data analysis

CHR and CFR were analysed in a fully Bayesian meta-analytic framework. A fully Bayesian framework provides a natural way of combining information from across multiple trials of different sizes and does not require any additional transformations.^34^^,^^35^ Additionally, Bayesian models provide insight into full posterior distributions for metrics of interest. For determinations of CHR and CFR, hospitalisations, and deaths, respectively were taken as binomially distributed from the number of reported cases. A random effects framework was fit where study specific effects were allowed, and an overall pooled effect was estimated. Heterogeneity was assessed using a Bayesian formulation of I^2^ as described by Higgins and Thompson.^36^ To account for the diverse temporal element of the case reporting and potential for high heterogeneity between study periods, subgroup random effects meta-analysis was conducted for temporally similar outbreak periods defined as pre-2017, 2017–2021, and 2022 and the pooled effects estimated. Weakly informative priors were used in all cases. Gelman-Rubin metrics were examined for proper chain mixing and effective sample sizes of the posterior distributions were examined to ensure that the posterior distribution had been sufficiently explored. All outcomes were reported as the median and 95% credible interval (CrI) of the posterior distributions. Mathematical details are available in the Appendix.

All analyses were conducted in R version 4.1.3 (2022-03-10) and Stan version 2.29.2.^37^

### Role of the funding source

There was no funding source for this study. All authors had full access to all the data in the study and had final responsibility for the decision to submit for publication.

## Results

**Fig. 1:**
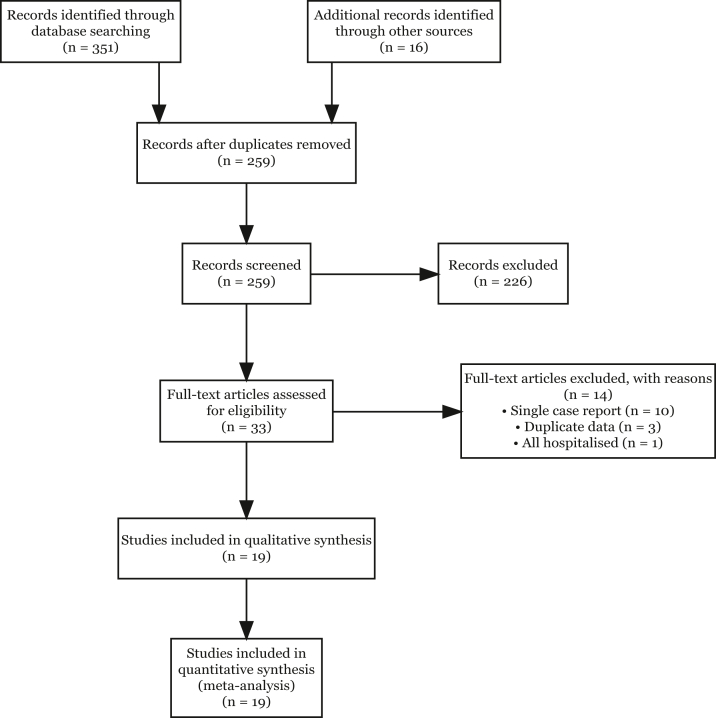
Study selection flowchart.

A total of 259 unique documents were identified by applying the search strategy and manually researching references lists and national/international data sources. Of those that were screened, 226 were excluded with the most common reason being that they did not report any case or hospitalization metrics, were different reports on the same outbreak, or were editorial commentaries. Of the remaining 33 documents reviewed in full, 11 documents were descriptions of single cases or of hospitalized patients only and were excluded. A further three documents were presentations of the same outbreak with duplicate data. Nineteen documents included information on cases and hospitalisations and were included in the systematic review and meta-analysis (Fig. 1).

Epidemiological information, treatments, and outcomes are documented in Table 1. A total of 7553 probable, suspected, and confirmed MPX cases were identified with a total of 555 reported hospitalisations. The median number of cases reported was 48 (interquartile range, IQR, 18–183) while the median number of reported hospitalisations was 7 (IQR, 3–17) as shown in Table 2. Reported deaths ranged from zero to eight with a median of zero (IQR, 0–0). The median age of cases was 34 (IQR, 29–38) based on 16 studies (n = 2010) where age was reported. Most of the cases were documented in males (98%, 7339/7489). Most studies originated in European nations (n = 9) followed by African nations (n = 7) and the United States of America (n = 2). Spain was the country of origin in four studies while the Central African Republic appeared in three studies. One study contained case information from individuals from 16 different countries on five continents. Nearly all of the studies included were retrospective analyses of prior or ongoing outbreaks of MPX, while one study^50^ was a case control study on the effect of HIV on MPX outcomes. The majority of studies reported confirmed cases (12/19, 63%), while six studies reported suspected, possible and confirmed cases (6/19, 32%), with one study reporting suspected cases only. Studies which met inclusion criteria ranged from 2003 to 2022 and both Clade I (2/19) and Clade II (9/20) clades were represented, though the clade was not fully specified in the plurality of documents analysed (8/19, 42%). Six, two, and eleven studies were considered parts of the pre-2017, 2017–2021, and 2022 outbreaks, respectively. There were no studies which met the inclusion criteria prior to 2003 due to a lack of hospitalisations being reported. More recent studies documented the 2022 outbreak included mostly men with fewer women and children compared to studies available prior to 2022. Many of the studies in 2022 specifically document sexual activity, where the majority of reported cases occur among individuals who identify as gay or MSM.^2^^,^^27^, ^28^, ^29^^,^^44^^,^^47^^,^^48^ When treatments were specified, the most often used pharmaceuticals were antibiotics but more recent documents indicate the use of antivirals (e.g., tecovirimat).^27^, ^28^, ^29^^,^^41^ Five of the studies explicitly documented an initial spillover event followed by human-to-human transmission.^15^^,^^38^^,^^39^^,^^41^ Sustained human-to-human transmission appears to be the most likely mode of transmission in most studies in 2022. A total of 15 deaths were recorded in four different studies. Seven of the recorded deaths were documented by three studies during outbreaks in the Central African Republic between December 2015 and January 2016. Of these seven deaths, five occurred in children five years old and younger. Two of these studies were by the same lead author.^39^^,^^40^ The remaining eight deaths were recorded by a single study between 2017 and 2019 in Nigeria. Of these eight deaths, three occurred in children less than 15 years old. In one study, the hospitalized patient outcomes were unavailable.^42^ Four studies were judged to have moderate or high bias due to questions regarding non-response (ascertainment of cases) and small sample sizes (Table 3).

**Table 1:** Studies selected for estimated case hospitalisation and case fatality rates.

Study	County	Clade	Study type	Dates	Participants	Definition	Transmission type	Treatment	Cases	Hospitalisations	Deaths	Risk of bias
CDC (2003)^15^	United States	Not specified	Retrospective	May–July 2003	Median age 28 years old (range 1–51 years old)	Suspected and PCR confirmed	Majority exposed to prairie dogs (possible animal to human transmission)	Not specified	71	18	0	Low
Learned et al. (2005)^20^	Republic of the Congo	Not specified	Retrospective	April–June 2003	Median age 8 years (Range 0–30+ years old),73% Male; none vaccinated against smallpox	Probable and confirmed	Possible animal-to-human followed by human-to-human up to 7 generations (6 passages)	Not specified	11	3	0	Moderate
Formenty et al. (2010)^38^	Sudan	Clade I	Retrospective	Sep 2005–Jan 2006	All patients <32 year (range 8 months–32 years); 52% women	Probable and confirmed	Suspected animal to human and 4 chains of human to human	Not specified	19	8	0	Moderate
Kalthan et al. (2018)^39^	Central African Republic	Not specified	Retrospective	August–October 2016	Median age 24 years (range 1–58 years); 53% male	Suspected and confirmed	Animal to human and human to human	Not specified	26	16	2	Low
Kalthan et al. (2016)^40^	Central African Republic	Not specified	Retrospective	Dec 2015–Jan 2016	Median age 29 years old (Range 5 months–41 years old)	Suspected and confirmed	Not specified	Not specified	12	10	3	High
Nakoune et al. (2017)^41^	Central African Republic	Clade I	Retrospective	Dec 2015–Jan 2016	Median age 27.5 years old (range 15 months–41 years old)	Confirmed	Animal to human followed by human to human	Oral antibiotics, IV antibiotics, tetracycline eye ointment, furosemide and oxygen in case of pulmonary oedema	10	7	2	Low
WHO Africa (2017)^42^	Nigeria	Clade II	Retrospective	Sep–Oct 2017	Not specified	Suspected	Not specified	Not specified	13	4		High
Català et al. (2022)^43^	Spain	Not specified	Retrospective	May–July 2022	Average age 38.7 (standard deviation 8.2). All males, 10% prior history of smallpox vaccination, 42% living with HIV (78)	Confirmed	Human to human	Not specified	185	4	0	Low
ECDC (2022)^44^	Various	Clade II	Retrospective	2022	Majority between 31 and 40 years old; 43.1% indicate MSM, 0.4% bisexual; 55.9% unknown or missing	Confirmed	Not specified	Not specified	5504	339	0	Low
Girometti et al. (2022)^29^	United Kingdom	Not specified	Retrospective	May 2022	All men, median age 41 years (IQR 34–45), all identify as MSM	Confirmed	Likely human to human	Four individuals received antibiotic treatment (two received a course of intravenous ceftriaxone and oral doxycycline, one received intravenous ceftriaxone and oral metronidazole, and one received oral doxycycline and antiviral therapy with tecovirimat) and analgesia.	54	5	0	Low
Minhaj et al. (2022)^45^	United States	Clade II	Retrospective		Average age 40 years (range 28–61), 11 involved in international travel in 21 days prior. 16/17 identify as MSM.	Confirmed	Human to human	Not specified	17	1	0	Low
Moschese et al. (2022)^46^	Italy	Not specified	Retrospective	May–July 2022	Not specified	Confirmed	Human to human	Bacterial superinfections, cidofovir, tecoviritmat, analgesics, Ceftriaxone, daptomycin	34	3	0	Low
Orviz et al. (2022)^47^	Spain	Clade II	Retrospective	2022	Median age 35 years (IQR 29–44); all men; 87.5% men who have sex with men	Confirmed	Human to human	Not specified	48	1	0	Low
Patel et al. (2022)^28^	United Kingdom	Not specified	Retrospective	May–July 2022	Median age 38 years (IQR 32–42); all men; 99.5% GBMSM	Confirmed	Human to human	Fentanyl for pain; paracetamol, ibuprofen, opioids, lidocaine gel, oral laxatives; co-amoxiclav and meropenem for bacterial infection; tecovirimat	197	20	0	Low
Perez Duque et al. (2022)^2^	Portugal	Clade II	Retrospective	April–May 2022	Median age 33 years (range 22–51 years); all male; 18/19 MSM	Suspected, probable, and confirmed	Human to human	Not specified	27	3	0	Low
Rodriguez et al. (2022)^48^	Spain	Clade II	Retrospective	May–July 2022	Median age 37; 98.9% male; 290 reported MSM, 6 reported heterosexual out of 332 reporting sexual contact	Confirmed	Human to human	Not specified	530	30	0	Low
Tarín-Vicente et al. (2022)^49^	Spain	Clade II	Retrospective	May–June 2022	37 years (IQR, 31–42); 97% (175/181) male; 92% gay, bisexual, MSM	Confirmed	Human to human	Treatment not specified for proctitis, tonsillitis, and bacterial skin abscess (38.7% required treatment)	181	2	0	Low
Thornhill et al. (2022)^27^	Various	Not specified	Retrospective	April–June 2022	Median age 38 (range 18–68); 527/528 men; 98% gay or bisexual men	Confirmed	Human to human	Cidofovir; Tecovirimat; Vaccinia immune globulin	528	70	0	Low
Yinka-Ogunleye et al. (2022)^50^	Nigeria	Clade II	Retrospective Case Cohort	2017–2019	Median age 31 years (IQR: 26–38). 71% males (n = 61). Included 6 children less than 15 years old.	Confirmed	Human to human	Not specified	86	11/58	8	Low

Abbreviations: CDC, Centers for Disease Control and Prevention; ECDC, European Center for Disease Control; GBMSM, gay, bisexual, and other men who have sex with men; IQR, interquartile range; IV, intravenous MSM, men who have sex with men; PCR, polymerase chain reaction.

**Table 2:** Characteristics of included subjects and studies.

Characteristic	N = 19
**Age (years, median, IQR)**	35 (28, 38)
Unknown	4
**Sex: male (n, %)**	7339/7416 (98)
Unknown	3
**Outbreak (k)**	
pre-2017	6 (32%)
2017–2021	2 (11%)
2022	11 (58%)
**County (k)**	
Central African Republic	3 (16%)
Italy	1 (5.3%)
Nigeria	2 (11%)
Portugal	1 (5.3%)
Republic of the Congo	1 (5.3%)
Spain	4 (21%)
Sudan	1 (5.3%)
United Kingdom	2 (11%)
United States	2 (11%)
Various	2 (11%)
**Clade (k)**	
Clade I	2 (11%)
Clade II	8 (42%)
Not specified	9 (47%)
**Cases per study (median, IQR)**	48 (18, 183)
**Hospitalisations per study (median, IQR)**	7 (3, 17)
**Deaths per study (median, IQR)**	0 (0, 0)
Unknown	1
**Definition (k)**	
Confirmed	12 (63%)
Probable and confirmed	2 (11%)
Suspected	1 (5.3%)
Suspected and confirmed	3 (16%)
Suspected, probable, and confirmed	1 (5.3%)
**Risk of bias (k)**^a^	
Low	15 (79%)
Moderate	2 (11%)
High	2 (11%)

a Assessment tool is adapted from the quality assessment tool developed by Hoy and colleagues and adapted by Werfalli and colleagues for studying prevalence in populations.

**Table 3:** Quality assessment scores.

Study	Representativeness of the sample	Sample size	Was the study's target population a close representation of the national population in relation to relevant variables?	Was the sampling frame a true or close representation of the target population?	Was some form of random selection used to select the sample, or was a census undertaken?	Was the likelihood of non-response bias minimal?	Were data collected directly from the participants (as opposed to a proxy)?	Was the same mode of data collection used for all participants?	Was the length of the shortest prevalence period for the parameter of interest appropriate?	Were the numerator (s) and denominator (s) for the parameter of interest appropriate?	Score	Risk of Bias
CDC (2003)^3^	∗	∗	∗	∗	∗	∗	∗	∗	∗	∗	10	Low
Learned (2005)^20^	∗				∗		∗	∗	∗	∗	6	Moderate
Formenty (2010)^38^	∗		∗	∗			∗	∗	∗	∗	7	Moderate
Kalthan (2016)^40^					∗		∗	∗	∗	∗	5	High
Nakoune (2017)^41^	∗			∗	∗	∗	∗	∗	∗	∗	8	Low
WHO Africa (2017)^42^	∗		∗	∗						∗	4	High
Kalthan (2018)^39^	∗	∗	∗		∗		∗	∗	∗	∗	8	Low
Català (2022)^43^	∗	∗	∗	∗		∗	∗	∗	∗	∗	9	Low
ECDC (2022)^26^	∗	∗	∗	∗	∗	∗	∗	∗	∗	∗	10	Low
Girometti (2022)^29^		∗	∗	∗	∗	∗	∗	∗	∗	∗	9	Low
Minhaj (2022)^45^	∗		∗	∗	∗		∗	∗	∗	∗	8	Low
Moschese (2022)^46^		∗	∗	∗	∗	∗	∗	∗	∗	∗	9	Low
Orviz (2022)^47^		∗	∗	∗	∗	∗	∗	∗	∗	∗	9	Low
Patel (2022)^28^	∗	∗		∗	∗	∗	∗	∗	∗	∗	9	Low
Perez Duque (2022)^2^		∗	∗	∗	∗	∗	∗	∗	∗	∗	9	Low
Rodriguez (2022)^48^	∗	∗	∗	∗	∗	∗	∗	∗	∗	∗	10	Low
Thornhill (2022)^27^	∗	∗	∗	∗	∗	∗	∗	∗	∗	∗	10	Low
Yinka-Ogunleye (2022)^50^	∗	∗	∗	∗	∗	∗	∗	∗	∗	∗	10	Low
Tarín-Vicente (2022)^49^		∗	∗	∗	∗	∗	∗	∗	∗	∗	9	Low

**Fig. 2:**
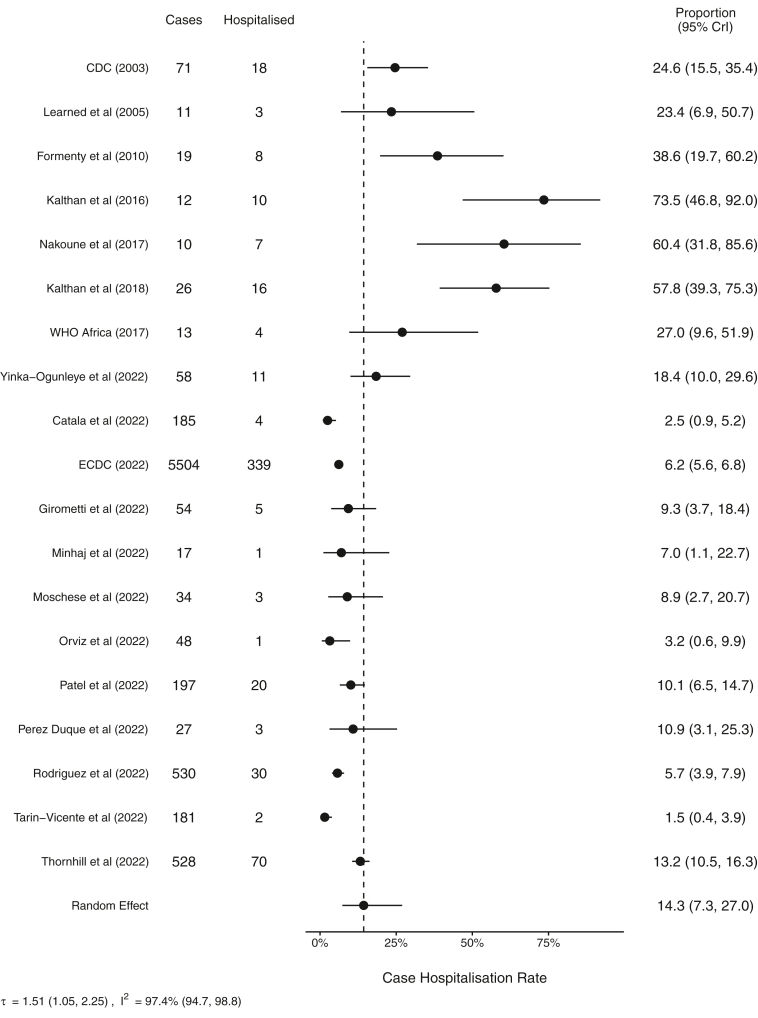
**Forest plot of resulting meta-analysis for case hospitalisation rates for all studies.** CrI, Bayesian credible interval.

**Fig. 3:**
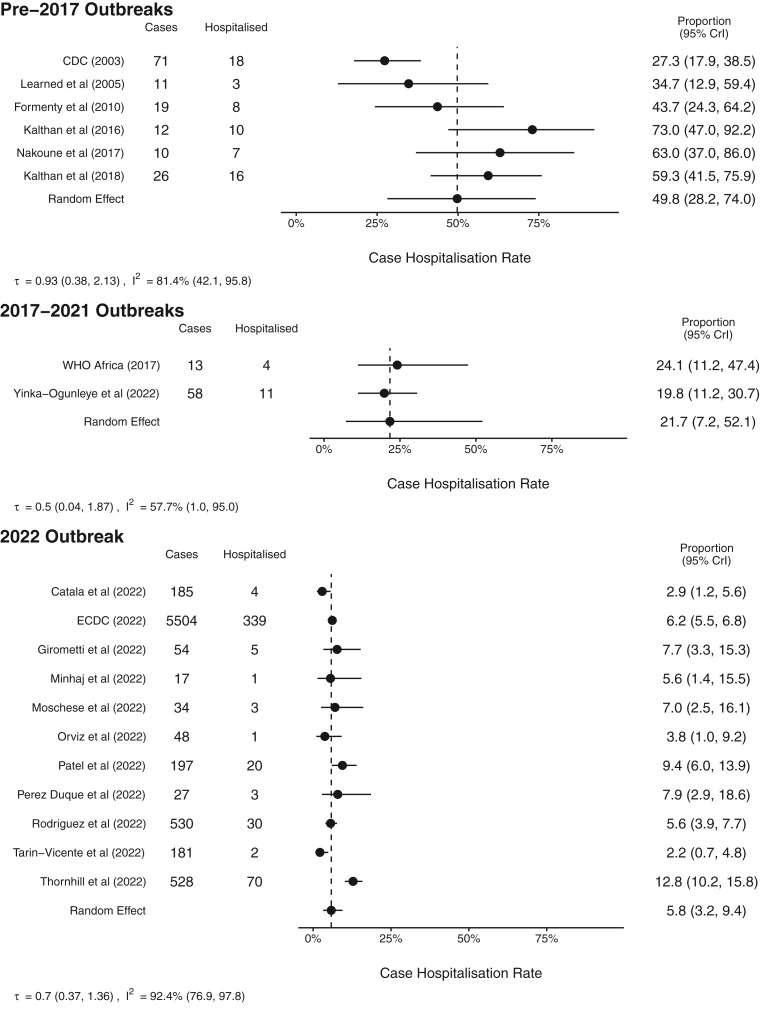
**Forest plot of resulting meta-analysis for case hospitalisation rates subgroup analysis by outbreak period.** CrI, Bayesian credible interval.

The combined CHR was estimated to be 14.3% (95% credible interval, CrI, 7.3–27.0, I^2^ 97.4%) (Fig. 2) with a high degree of heterogeneity between studies. Individual study CHR estimates ranged from 1.5% (0.4–3.9) in the study by Tarín-Vicente and colleagues^49^ to as high as 73.5% (46.8–92.0) in the study by Kalthan and colleagues and showed a strong temporal trend (Fig. 2).^39^ Further subgroup analysis by outbreak period resulted in estimated CHRs of 49.8% (28.2–74.0, I^2^ 81.4%), 21.7% (7.2–52.1, I^2^ 57.7%), and 5.8% (3.2–9.4, I^2^ 92.4%) during the pre-2017, 2017–2021, and 2022 outbreaks, respectively (Fig. 3), with high levels of heterogeneity indicating these pooled estimations should be interpreted with caution. An additional sensitivity analysis was conducted on those studies with 100 or more reported cases representing six studies during the 2022 outbreak yielding a random effect estimated CHR of 5.3% (2.0–11.8, I^2^ 95.6%) with a high level of heterogeneity (see Appendix Figure S1). Furthermore, an additional sensitivity analysis was conducted only on those studies reporting confirmed cases during the 2022 outbreak and a similar pooled estimate was found with an estimated CHR of 5.5% (2.9–9.4, I^2^ 93.4% (see Appendix Figure S2). High levels of heterogeneity were observed in both sensitivity analyses indicating that results should be interpreted with caution.

**Fig. 4:**
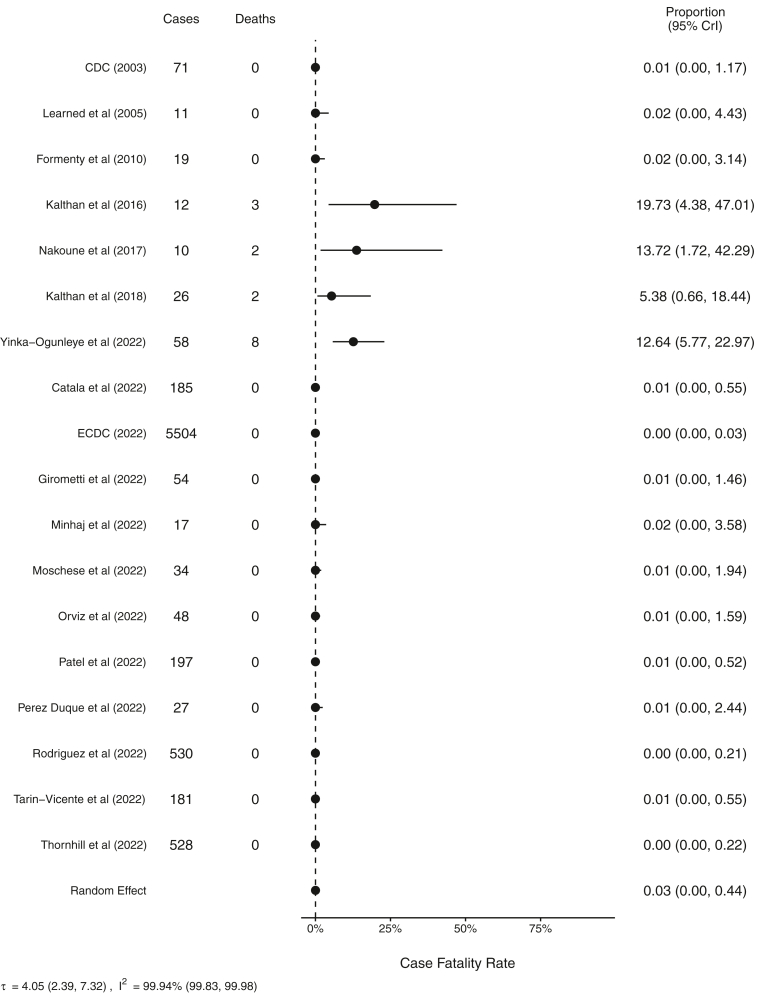
**Forest plot of resulting meta-analysis for case fatality ratios for all studies.** CrI, Bayesian credible interval.

There were a total of 15 recorded deaths and 7540 recorded cases where information on patient outcomes was available. Deaths were observed in four of the studies. Pooled CFR including all studies where deaths were recorded was estimated to be 0.03% (95% CrI 0.00–0.44, 99.9%) in the random effects model (Fig. 4). However, there was a high degree of heterogeneity Availability of genetic clade information and recorded deaths limited analysis for clade specific CFR. In studies meeting in conclusion criteria, there were no recorded deaths during the 2022 outbreak.

## Discussion

Our study suggests that 14.3% (7.3–27.0) of cases may require hospitalization, with a higher degree of uncertainty and heterogeneity based on setting and outbreak period and these results should be interpreted with caution. Further subgroup analysis indicates that the expected CHR during the 2022 outbreak is 5.8% (3.2–9.4). In a study of smallpox in Europe between 1950 and 1971, 85% (35/41) import cases sought the care of physician^51^ suggesting a potential six-fold increase in hospitalization compared to MPX; however, direct comparisons of MPX CHR against smallpox are challenging given the general awareness of potential poor outcomes with smallpox infections as well as advances in medical care. Another more common viral infection presenting with a rash is varicella zoster virus (VZV) infection, a herpes virus, where 1–14 hospitalisations would be expected per 1000 reported cases depending on patient age.^52^ The estimate MPX CFR of 0.03% (95% CrI 0.00–0.44) would suggest 0.3 to four deaths per 1000 cases. This CFR is roughly 20–100 times greater than that for reported VZV infections but remains 50–1500 times less than that of variola minor (1% CFR) or variola major (30% CFR).^53^ However, there is a high degree of heterogeneity in studies and more information is needed in order to better estimate this value. Overall, this review found no published literature on case hospitalization rates prior to 2003 and all studies were retrospective in nature, reflecting a dearth of literature on this subject.

Early estimates for CFR estimated a clade specific rate between one and ten percent.^14^ However, many of the studies used in the estimation of these CFRs did not provide information regarding the number of hospitalisations and could be a reflection of the heterogeneity of care and availability of treatments to infected individuals.^54^ All of the studies included in this analysis concerning the 2022 outbreak have been set in upper middle to high income countries, again reflecting a potential difference in CHR and CFR due to access to care. There is also some evidence that CFR may have a strong age gradient. The 2022 outbreak of MPX infections has been predominantly identified in young and middle-aged adult men where morbidity and mortality historically have been lower. Prior outbreaks show a high degree of mortality in younger populations.^40^^,^^41^^,^^50^ For instance, Kalthan and colleagues found that out of ten recorded hospitalisations, all three deaths occurred in children less than 10 years old.^40^ Similarly, the two deaths reported by Nakoune and colleagues were among children aged 15 months and five years old.^41^ In a systematic review on MPX epidemiology from the 1970s to 1999, children aged less than 10 years accounted for 100% of the total 47 reported deaths whereas data from 2000 to 2019, fatalities occurred in only 37.5% (6/16) of children less than 10 years old.^55^ In the 2003 US outbreak, a risk factor for severe disease requiring intensive care was being a paediatric patients (aged less than 18 years).^56^ Jezek and colleagues’ 1987 study of 282 MPX patients observed all deaths in those less than ten years old,^57^ however, no information regarding availability of care or hospitalisations were noted. Similarly, of the 15 deaths noted in this study, eight were reported in children 15 years old or younger, five of which were in children younger than five years.^39^, ^40^, ^41^^,^^50^ Many of the studies documenting the 2022 outbreak do not reflect any paediatric cases, which may have an influence on the estimated CFR. Furthermore, CFR is not only determined by the pathogen, but also what resources are available to treat the infected individuals. Our analysis considers the CFR where death and hospitalization records were both available which should better represent the expected rates when medical care is available. No deaths were reported in a recent international case series across 16 countries^27^ and in an observational study from the United Kingdom.^29^

While the current 2022 global outbreak has been predominantly identified in younger men, often MSM, transmission of MPX infections to pregnant women, children, immunocompromised individuals, and older individuals may increase the observed morbidity and mortality in the current outbreak. As human-to-human transmission is thought to occur from prolonged skin-to-skin contact or exposure to respiratory droplets, transmission from caregivers to children and between children is a major concern. This mode of transmission among family contacts and caregivers to individuals with MPX infections has been observed in prior outbreaks and case reports.^41^^,^^58^ While the current outbreak is concentrated in young MSM, introduction of MPX into other groups through household contacts could result in changes in CHR. With the declaration of smallpox eradication and cessation of smallpox vaccination, which provides notable cross protection against MPX,^59^ most individuals born after 1980, and even earlier in some countries, may not have not been vaccinated against smallpox so new introductions of MPX would be in a largely immune naïve population.^22^ Those individuals who were vaccinated against smallpox (e.g., military personnel) would likely have waning immunity against infection. A systematic review report that 80–96% of MPX cases occur among individuals unvaccinated against smallpox,^55^ which suggests the role that vaccination could play in reducing onward transmission.

Effects of treatment and supportive care for individuals infected with MPX remains an open question and plays an important role in defining the CHR and CFR. Effective treatments may reduce both the likelihood of hospitalization and mortality. We documented the use of antibiotics to treat secondary bacterial infections in four studies,^27^, ^28^, ^29^^,^^41^ another complication of MPX infections, which represents further contribution to the use of antimicrobials and the prospect of antimicrobial resistant organisms. Tecovirimat, an antiviral, is available under an expanded use protocol in the United States and under additional monitoring by the European Medicines Agency, but effectiveness of tecovirimat in treating MPX (i.e., reducing disease duration or rates of complications) is not well characterized.^3^^,^^60^ Similar questions exist regarding the effectiveness of Cidofovir and Brincidofovir.^61^ More rapid recognition and treatment of MPX infections could contribute to reductions in both CHR and CFR, especially through the timely use of antivirals which may reduce the length of infection, likelihood of hospitalization, and reduce opportunities for secondary infections due to a shortened disease course. Documented therapies for pain management have included use of opioids as well as non-opioid analgesics.^27^, ^28^, ^29^ Concerns has been raised about the use of opioids given the issues of addiction and potential for constipation which may further exacerbate proctitis associated with MPX,^27^^,^^28^ but no complications were noted in the literature reviewed therefore the potential implication on CHR remains unclear.^28^ More research will be needed in order to fully understand the effectiveness of these treatment options for MPX and their impact on CHR and CFR.

Early studies of MPX CHR and CFR have primarily been limited to outbreak investigations in countries where MPX is considered endemic, with hospitalization rates often not reported.^62^^,^^63^ Prior to the 2022 global outbreak of MPX, most infections outside of Africa, when detected were immediately quarantined. Thus, inferences regarding case hospitalization ratios have been limited.^64^^,^^65^ However, as sustained human-to-human transmission has been observed and the number of cases grow, it will be important to quantify the morbidity and mortality of MPX infections and the potential for CHR and CFR to evolve. Formenty and colleagues remarked as early as 2010 when writing about a 2004 outbreak in Sudan regarding the possibility of cryptic, sustained human-to-human transmission and community spread of infection with lower CFR, reflecting potential host adaptation.^38^ As the general incidence increases, there are increased opportunities for viral evolution and adaptation to human hosts, with early studies suggesting human adaption during the current outbreak.^66^ Several studies indicate between 41 and 67% of those infected with MPX are living with human immunodeficiency virus (HIV) which may not only have an impact on morbidity and mortality, but also within-host adaptation and potential changes in disease severity due to potential decreased immune function in this population, and thus associated CHR and CFR.^27^^,^^29^ A retrospective review hospitalized MPX patients during the 2017–2018 Nigeria outbreak has suggested that when compared to those who do not have HIV, patients with HIV have worse outcomes with higher rates of secondary bacterial infections and larger legions when infected with MPX.^67^ Another retrospective cohort study has found that those persons living with HIV were nearly 14 times more likely to die when infected with MPX,^50^ however CD4+ cell counts were unavailable leaving an open question how cell counts may impact MPX outcomes. The high rates of concurrent sexually transmitted infections such as HIV and gonorrhoea in the 2022 outbreak, and this too may have an impact on the morbidity and mortality amongst those infected and should continue to be monitored.^27^^,^^29^

This systematic review and meta-analysis are subject to several limitations. There is a high degree of heterogeneity in the studies meeting our inclusion criteria with no studies prior to 2003. Access to care and hospital seeking behaviours are likely heterogenous across the different studies and associated countries and likely contribute to the observed heterogeneity in the studies. Our definition of hospital care being available based on the report of any hospitalisations risks over-stating the CHR. Availability of care rather than true need may influence CHR in some settings. Similarly, the level of care that could be provided may differ across facilities depending on resources, as would clinical requirements for a hospital admission. For instance, several studies in 2022 reported providing broad spectrum antibiotics, analgesics, and antivirals, while Nakoma and colleagues reported that one patient was transferred to a different hospital due to a lack of antibiotics.^41^ Furthermore, we included all case definitions in our analysis (suspected, probable, and confirmed) which could introduce bias. All studies are subject to case ascertainment bias where potential infection may not be identified or only close contacts tested for evidence of MPX rather than broader surveillance. Ascertainment bias is especially a risk in retrospective studies and outbreak investigations. Broad serosurveillance surveys are unavailable which could provide better insight into the true prevalence of MPX and the level of immunity in different countries. Additionally, this study does not consider potential for asymptomatic or pauci-symptomatic disease transmission, which would reflect an undercount of potential cases but could have a role in onward transmission. Information regarding the genetic clade was not available for all the available documents and could provide deeper insight into host adaption and ongoing evolution in the context of community spread in the 2022 outbreak.

Based on this systematic review and meta-analysis we find that the random effects CHR of monkeypox infection is estimated to be 14.3% (7.3–27.0) and 5.8% (3.2–9.4) in the 2022 outbreak subgroup, however random effects models show that there is a high degree of heterogeneity based on case identification and access to treatment. Similarly, the CFR when hospital care is available is estimated to be 0.03% (0.00–0.44), again with a high degree of heterogeneity based on access to care and the age of infected individuals. Future analyses should consider additional sources of heterogeneity which could include age, case ascertainment, and comorbidities which have not been well documented prior to the 2022 outbreak. Robust estimates of expected CHR and CFR can be used to estimate actual prevalence of infection as deaths and hospitalisations are more likely to be recorded in government statistics. As the current 2022 global outbreak unfolds, these numbers will be further refined and public health interventions such as widespread education, vaccination, and access to treatment are expanded.

## Contributors

MD and JS conceived the study and wrote the initial protocol. MD, JS, AS, CM reviewed the literature. MD and JW screened the literature for inclusion and collected the data. MD and JW assessed the texts for risk of bias. MD conducted the statistical analysis with input from JS. All authors had access to the underlying data and data were verified by MD, JW, and JS. All authors contributed to interpretation of the study results. MD drafted the initial manuscript with critical revisions from CP, JW, and AS. All authors contributed to the final draft. All authors approved the final version submitted for publication.

## Data sharing statement

All data used in this analysis is available in the manuscript.

## Declaration of interests

CJM acknowledges support from BINX, 10.13039/100012399Biomedical Advanced Research and Development Authority, 10.13039/100004330GlaxoSmithKline, Becton Dickinson, United States Center for Disease Control and Prevention, 10.13039/100017037Cepheid, Gilead, 10.13039/100015160Hologic, Lupin, National Association of County and City Health Officials, and 10.13039/100000002National Institutes of Health paid to her employer.
