# Supplementary material for: Global monkeypox case hospitalisation rates: A rapid systematic review and meta-analysis
Source: eClinicalMedicine. 2022 Oct 31;54:101710. doi: 10.1016/j.eclinm.2022.101710 (PMC9621693; doi:10.1016/j.eclinm.2022.101710)
Supplement: Appendix [file mmc1.pdf]

Appendix
Search phrase used

Table S1: Database and search terms used in systematic review and meta-analysis

**Database**	**Search Term**
PubMed	((monkeypox[title] OR "monkey pox"[title] OR "Monkeypox"[Mesh] OR "Monkeypox virus"[Mesh]) AND ("HOSPITAL" OR "HOSPITALIZATION" OR "HOSPITALISATION") AND ("1950/01/01"[dp] : "2022/08/02"[dp]) NOT review[pt])
Embase	('monkeypox'/exp OR 'monkeypox' OR 'monkey pox'/exp OR 'monkey pox') AND ('hospitalization'/exp OR 'hospitalization' OR 'hospital'/exp OR 'hospital' OR 'hospitalisation')
MedRxiv	"(Monkeypox) AND (Hospitalization OR Hospitalisation)" (match whole all) and posted between "01 Jan, 1900 and 02 Aug, 2022"
Preprints with The Lancet	Monkeypox

Risk of Bias Assessment

External validity (1 Point Each)

1. Was the study's target population a close representation of the national population in relation to relevant variables?
2. Was the sampling frame a true or close representation of the target population?
3. Was some form of random selection used to select the sample, OR was a census undertaken?
4. Was the likelihood of non-response bias minimal?

Internal Validity (1 Point Each)

1. Were data collected directly from the participants (as opposed to a proxy)?
2. Was an acceptable case definition used in the study?
    a. Clear definition of a case (e.g., suspected, probably, laboratory confirmed)
3. Was the study instrument that measured the parameter of interest shown to have validity and reliability?
4. Was the same mode of data collection used for all participants?
5. Was the length of the shortest prevalence period for the parameter of interest appropriate?
6. Were the numerator(s) and denominator(s) for the parameter of interest appropriate?

Total Points (10)

This assessment tool is adapted from the quality assessment tool developed by Hoy and colleagues[1] and adapted by Werfalli and colleagues.[2]

Mathematical Model

Random Effects Estimate

$$y_j \sim Binomial(k_j, \theta_j)$$

Where:

$$logit(\theta_j) = \mu + \nu_j$$

$$\nu \sim normal(0, \sigma)$$

$$\sigma \sim exponential(1)$$

Where y represents hospitalisations or deaths, k represents the number of cases, and theta represents the probability of death or hospitalization for a given trial, j as per Siegel and colleagues.[3] Further, a Bayesian implementation of heterogeneity, I^2, was calculated following the method proposed by Higgins and Thompson for Bayesian models.[4]

References

1. Hoy D, Brooks P, Woolf A, et al. Assessing risk of bias in prevalence studies: modification of an existing tool and evidence of interrater agreement. *Journal of Clinical Epidemiology*. 2012;65(9):934-939. doi:10.1016/j.jclinepi.2011.11.014

2. Werfalli M, Musekiwa A, Engel ME, Ross I, Kengne AP, Levitt NS. The prevalence of type 2 diabetes mellitus among older people in Africa: a systematic review study protocol. *BMJ Open*. 2014;4(6):e004747. doi:10.1136/bmjopen-2013-004747

3. Siegel L, Rudser K, Sutcliffe S, et al. A Bayesian Multivariate Meta-Analysis of Prevalence Data. *Stat Med*. 2020;39(23):3105-3119. doi:10.1002/sim.8593

4. Higgins JPT, Thompson SG. Quantifying heterogeneity in a meta-analysis. *Statistics in Medicine*. 2002;21(11):1539-1558. doi:10.1002/sim.1186

Figure S1: Forest plot of result meta-analysis for case hospitalization rates for those studies with more than 100 cases reported. CrI = Bayesian credible interval.

Figure S2: Forest plot of result meta-analysis for case hospitalization rates for those studies with only confirmed cases reported. CrI = Bayesian credible interval.

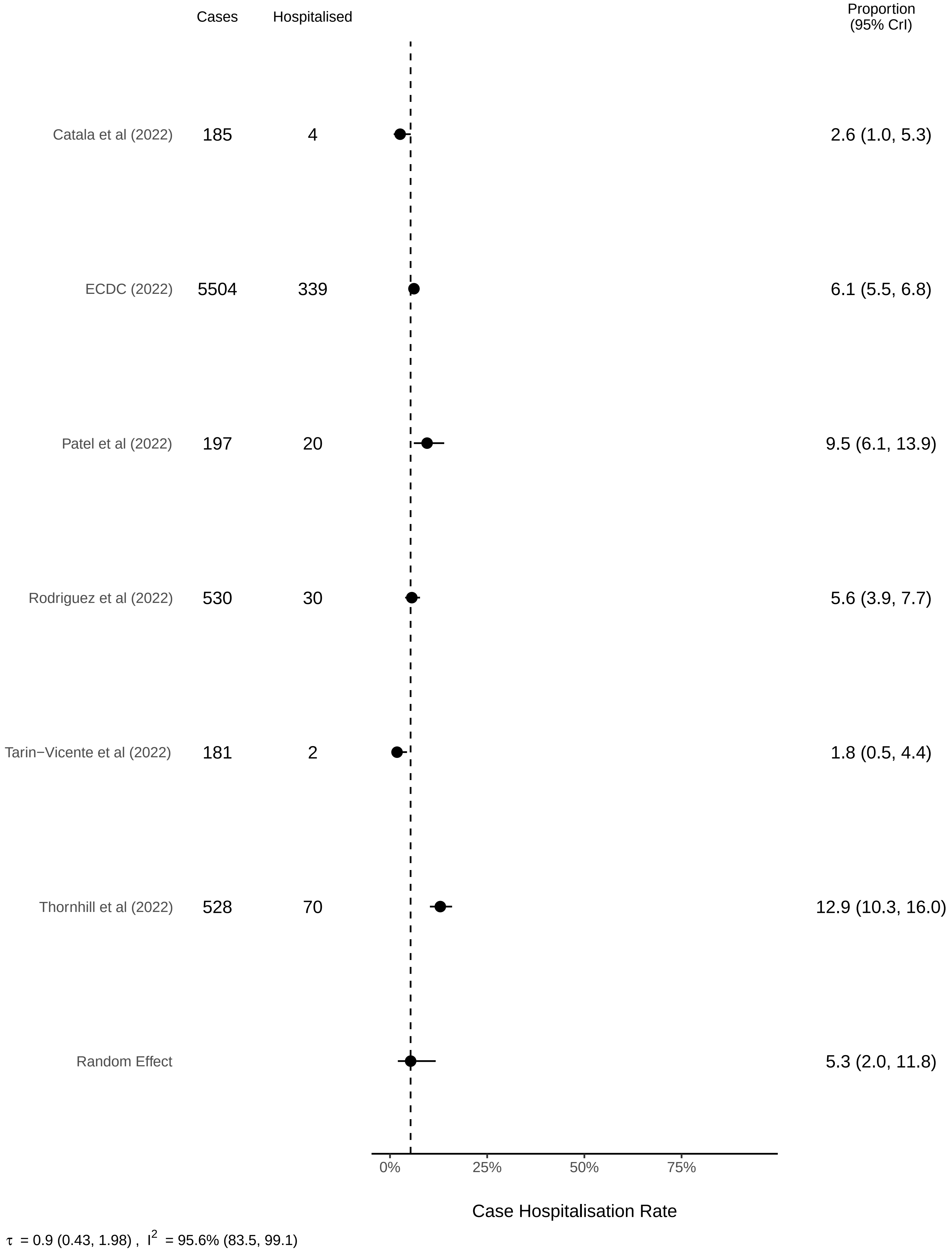
Cases
Hospitalised
Proportion (95% CrI)
Catala et al (2022) 185 4 2.6 (1.0, 5.3)
ECDC (2022) 5504 339 6.1 (5.5, 6.8)
Patel et al (2022) 197 20 9.5 (6.1, 13.9)
Rodriguez et al (2022) 530 30 5.6 (3.9, 7.7)
Tarin−Vicente et al (2022) 181 2 1.8 (0.5, 4.4)
Thornhill et al (2022) 528 70 12.9 (10.3, 16.0)
Random Effect 5.3 (2.0, 11.8)
0%
25%
50%
75%
Case Hospitalisation Rate
τ = 0.9 (0.43, 1.98) , I2 = 95.6% (83.5, 99.1)

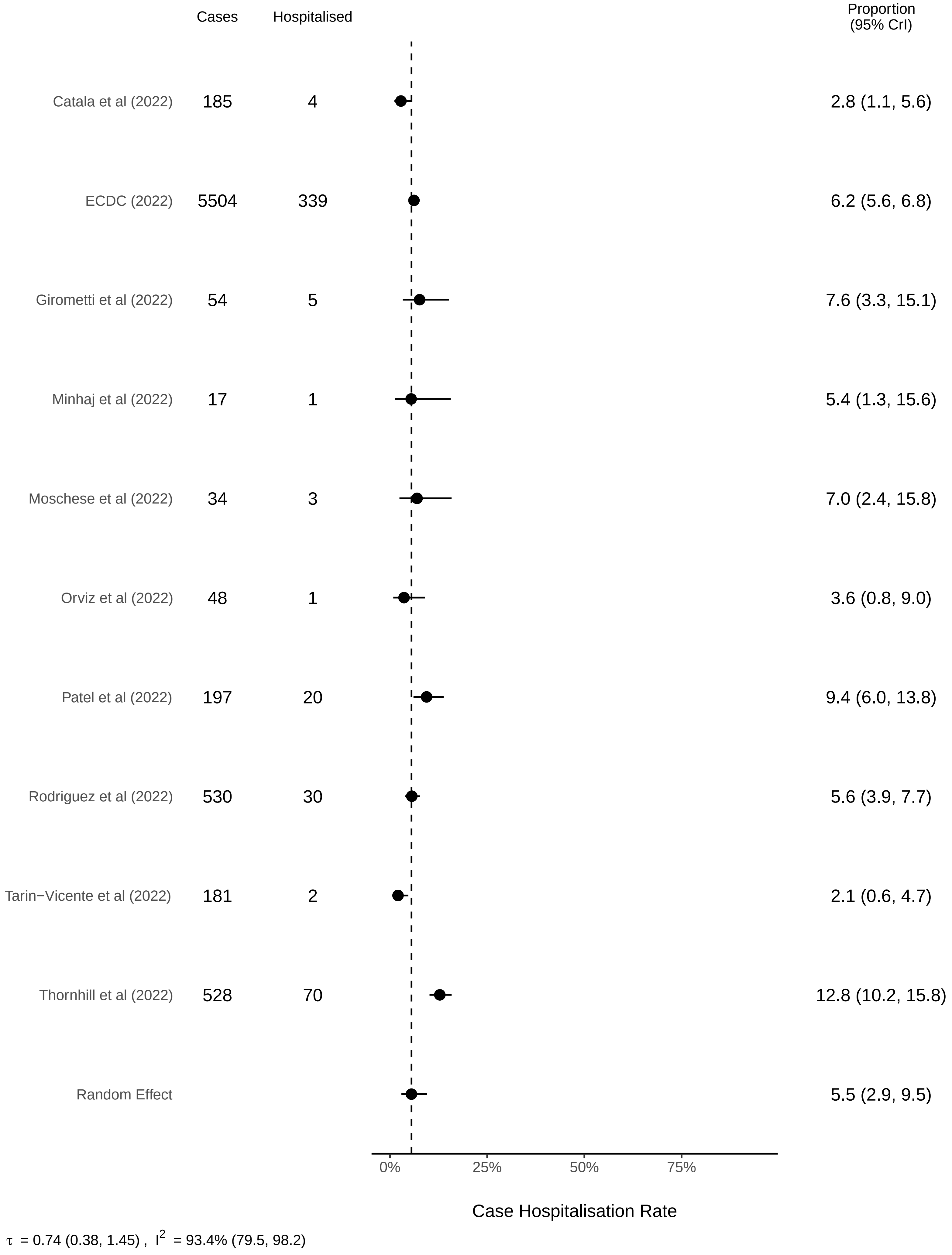
Cases
Hospitalised
Proportion
(95% CrI)
Catala et al (2022)
185
4
2.8 (1.1, 5.6)
ECDC (2022)
5504
339
6.2 (5.6, 6.8)
Girometti et al (2022)
54
5
7.6 (3.3, 15.1)
Minhaj et al (2022)
17
1
5.4 (1.3, 15.6)
Moschese et al (2022)
34
3
7.0 (2.4, 15.8)
Orviz et al (2022)
48
1
3.6 (0.8, 9.0)
Patel et al (2022)
197
20
9.4 (6.0, 13.8)
Rodriguez et al (2022)
530
30
5.6 (3.9, 7.7)
Tarin−Vicente et al (2022)
181
2
2.1 (0.6, 4.7)
Thornhill et al (2022)
528
70
12.8 (10.2, 15.8)
Random Effect
5.5 (2.9, 9.5)
0%
25%
50%
75%
Case Hospitalisation Rate
τ = 0.74 (0.38, 1.45) , I2 = 93.4% (79.5, 98.2)
